# Exclusive destruction of mitotic spindles in human cancer cells

**DOI:** 10.18632/oncotarget.15343

**Published:** 2017-02-15

**Authors:** Leonid Visochek, Asher Castiel, Leonid Mittelman, Michael Elkin, Dikla Atias, Talia Golan, Shai Izraeli, Tamar Peretz, Malka Cohen-Armon

**Affiliations:** ^1^ The Neufeld Cardiac Research Institute, Department of Physiology and Pharmacology, Sackler School of Medicine, Tel-Aviv University, Tel-Aviv 69978, Israel; ^2^ Cancer Research Center, Sheba Medical Center, Ramat Gan 53621, Israel; ^3^ The Imaging Unit, Sackler School of Medicine, Tel-Aviv University, Tel-Aviv 69978, Israel; ^4^ Sharett Oncology Institute, Hadassah Medical Center, Ein-Kerem, Jerusalem 91120, Israel; ^5^ The Department of Human Molecular Genetics and Biochemistry, Sackler School of Medicine, Tel-Aviv University, Tel-Aviv 69978, Israel; ^6^ Sagol School of Neuroscience, Tel-Aviv University, Tel-Aviv 69978, Israel

**Keywords:** human cancer cells, mitotic spindles, NuMA, kinesins, phenanthrenes

## Abstract

We identified target proteins modified by phenanthrenes that cause exclusive eradication of human cancer cells. The cytotoxic activity of the phenanthrenes in a variety of human cancer cells is attributed by these findings to post translational modifications of NuMA and kinesins HSET/kifC1 and kif18A. Their activity prevented the binding of NuMA to α-tubulin and kinesins in human cancer cells, and caused aberrant spindles. The most efficient cytotoxic activity of the phenanthridine PJ34, caused significantly smaller aberrant spindles with disrupted spindle poles and scattered extra-centrosomes and chromosomes. Concomitantly, PJ34 induced tumor growth arrest of human malignant tumors developed in athymic nude mice, indicating the relevance of its activity for cancer therapy.

## INTRODUCTION

Small molecules acting as PARP1 inhibitors are tested in the clinic for their possible potency to promote cell eradication after irradiation or DNA damaging chemotherapy [1, 2]. The idea underlying their use for cancer therapy is based on the role of activated PARP1 in DNA breaks repair [3]. PARP1 activity is inhibited by molecules mimicking the binding of its substrate, NAD (nicotine amide-di-nucleotide) to the catalytic site [3, 4]. Interestingly, our recent studies disclosed a group of small phenanthrene molecules that exclusively eradicate human cancer cells. Despite acting as PARP1 inhibitors, their cytotoxic effect is not attributed to their PARP1 inhibition potency [6–8]. These small molecules exclusively eradicate human cancer cells during mitosis without impairing normal proliferating cells [6–8]. They share this activity with other phenanthrenes [9], but not with non-phenanthrene potent PARP1 inhibitors [1–3, 7–8]. Here, we identified target proteins modified by these phenanthrenes, which include the derivatives PJ34, Phen and Tiq-A [7]. As other PARP1 inhibitors, PJ34, Phen and Tiq-A rescue normal quiescent cells from cell-death under stress conditions (e.g. ischemia, or inflammation [4, 10]). However, in human cancer cells they cause an opposite effect, exclusively causing cell death during mitosis [6–9].

We found that PJ34, Phen and Tiq-A modify kinesins (HSET/kifC1 and kif18A) and NuMA (nuclear mitotic apparatus protein) in a variety of human cancer cells. As a result, NuMA binding to the kinesins and to α-tubulin (a main component in the microtubules) was abolished. Most significantly, spindles, spindle poles, bi-focal centrosomes clustering and chromosomes assembly and alignment in the spindle mid-zone were disrupted.

Recent studies indicated that NuMA transfer along the microtubules, and the activity of kinesins HSET/kifC1 and kif18A are necessary for spindle constraction in mitosis. The kinesin HSET/kifC1 (microtubules minus end-directed motor protein) sorts microtubules into bundles that construct the spindle. Its indispensable activity for spindle construction in human malignant cells has been reported [16–22]. The kinesin Kif18A moves towards the microtubule plus end [20, 22, 23]. Kif18A is apparently implicated in microtubules de-polymerization, necessary for chromosomes assembly in the spindle mid-zone. Loss of its function results in the formation of long microtubules and scattered chromosomes [23].

The non-motor protein NuMA has a microtubules-binding capacity, which is implicated in the formation of microtubule bundles, and in converging and tethering spindle microtubules ends to create stable spindle poles [11–13]. Its capacity to bind proteins proved necessary for its activity [11, 12, 14]. NuMA forms filamentous polymers, which are useful in transferring NuMA along the microtubules in both malignant and normal proliferating cells [12]. Its activity is controlled by phosphorylation (including phosphorylation by Pim1) and polyADP-ribosylation [13, 14]. PolyADP-ribosylation of NuMA by activated tankyrase1, which exists in malignant cells but hardly exists in normal somatic cells, facilitates its binding to proteins [14], including those anchored to the spindle poles [15]. In addition, NuMA transfer towards the spindle poles has been associated with its binding to the microtubules minus end-directed motor protein, dyneine [11–13].

We found that the phenanthrenes, PJ34, Phen and Tiq-A, prevent the post-translational modifications of NuMA, HSET/kifC1 and Kif18A exclusively in human cancer cells. Their activity impaired the binding of NuMA to the kinesins and to α-tubulin, a component in the microtubules (microtubules are polymers consisting of α-, β-tubulin heterodimers [24]). Concomitantly, spindles and spindle poles were disrupted, extra centrosomes in multi-centrosomal cancer cells were not clustered, and chromosomes were not aligned in the spindle mid-zone. These impairments that cause cell death during mitosis [6–8, 25], may underlie tumor growth arrest of human cancer tumors treated with PJ34.

## RESULTS AND DISCUSSION

Our search for proteins affected by the phenanthrenes PJ34, Tiq-A and Phen identified proteins implicated in mitosis. PJ34, Tiq-A and Phen affected the post translational modification of these target proteins, as indicated by the shift in their isoelectric points (pI). Changes in the pI of proteins indicate their covalent modifications [26]. Previously, we used this marker to identify activation of G-proteins and polyADP-ribosylation of nuclear proteins [27, 28].

**Figure 1:**
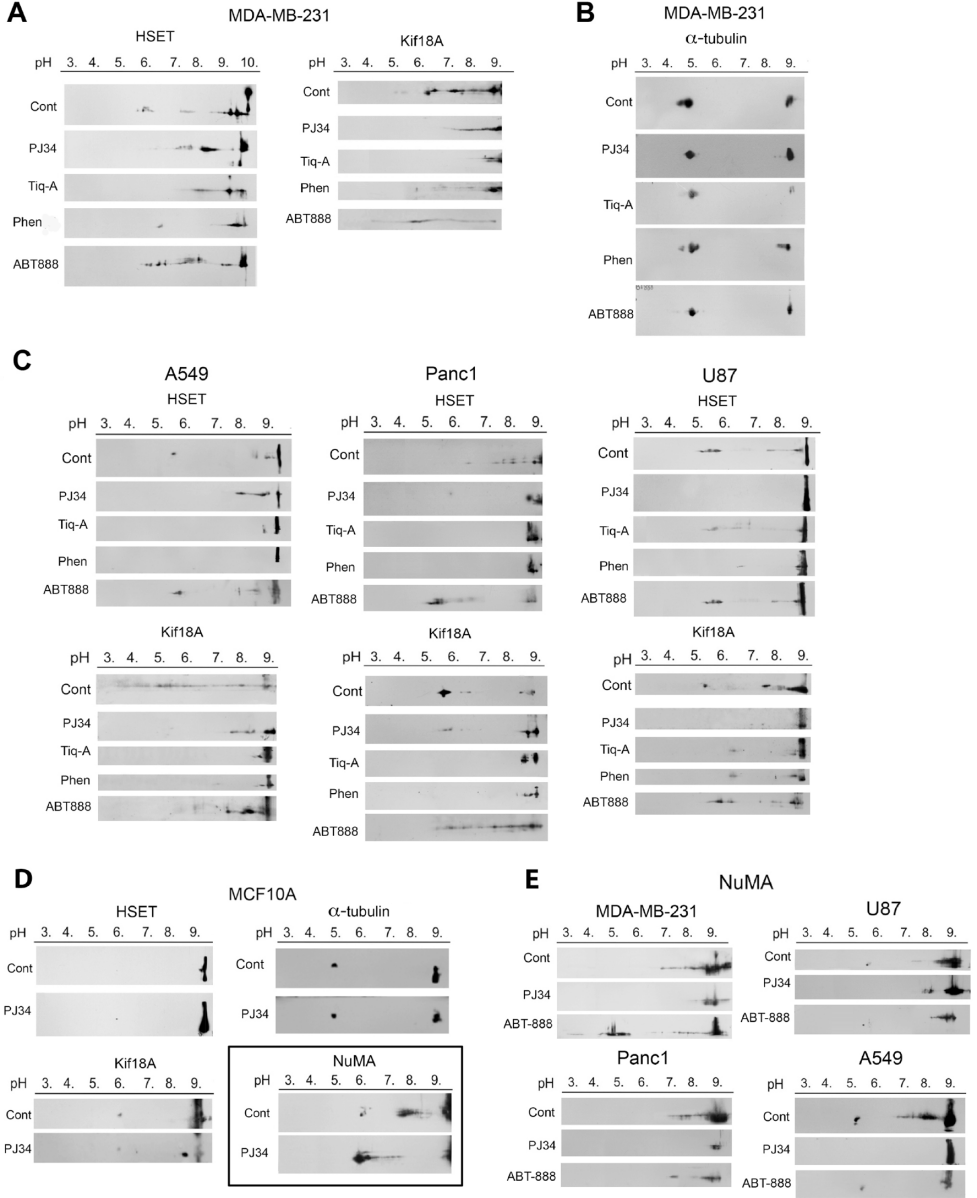
Post-translational modifications of kinesins and NuMA identified by changes in their isoelectric point in human cells treated with phenanthrenes (**A**) The isoelectric point of kinesins HSET/kifC1 and kif18A in human triple negative breast cancer cells MDA-MB-231 was shifted towards higher pH values (less negatively charged protein) after 27 hours incubation with the phenanthrene derivatives PJ34 (20 μM), Tiq-A (50 μM) or Phen (50 μM). No similar effect was induced by the non-phenanthrene PARP1 inhibitor ABT888 (20 μM). (**B**) The isoelectric point of α-tubulin was not affected by the phenanthrenes or by ABT888 in MDA-MB-231 cells. (**C**) The isoelectric point of kinesins HSET/kifC1 and kif18A was shifted towards higher pH values (less negatively charged protein) in human lung (A549), pancreas (PANC1) and glioblastoma (U87) cancer cells incubated for 27 h with PJ34 (20 μM), Tiq-A (50 μM) or Phen (50 μM). No similar effect on their isoelectric point was induced by the non-phenanthrene PARP1 inhibitor ABT888 (20 μM). (**D**) Treatment with the phenanthridine PJ34 (20 μM, 27 h) did not affect the isoelecrtic point of α-tubulin and kinesins HSET/kifC1 and Kif18A in human normal breast epithelial cells MCF10A. The isoelectric point of NuMA was shifted towards lower pH (more negatively charged protein) in these cells. (**E**) Treatment with PJ34 (20 μM, 27 h) shifted the isoelectric point of NuMA towards higher pH values (less negatively charged protein) in human malignant cells, including: breast (MDA-MB-231), lung (A549), pancreas (PANC1) and glioblastoma (U87) cells. Representative results of 4 different experiments in each cell type are displayed in A–E.

The phenanthrenes PJ34, Tiq-A and Phen shifted the pI of their target proteins towards higher pH values (less negatively charged protein) in a variety of human cancer cells, but not in normal proliferating cells. In cancer cells treated with the phenanthrenes (27 h incubation, 24 h after seeding), the shift in pI indicated an induced inhibition of their negatively charging covalent modifications (e.g., phosphorylation, ADP- and polyADP-ribosylation) (Figure 1).

Similar pI values of kinesins HSET/kifC1 and kif18A and the non-motor protein NuMA were measured in untreated normal and malignant cells. The phenanthrenes did not alter the pI of HSET/kifC1 and kif18A in normal epithelial cells MCF10A. NuMA was even more negatively charged in these proliferating cells after treatment (Figure 1D). However, in a variety of cancer cells, PJ34, Phen and Tiq-A prevented or attenuated the modifications of NuMA and kinesins HSET/kifC1 and kif18A (Figure 1A, 1C, 1E). Notably, the pI of these proteins was not affected by non-phenanthrenes acting as potent PARP1 inhibitors (e.g., ABT888), excluding possible implication of PARP1 inhibition in the modification of NuMA and kinesins by the phenanthrenes.

In addition, the phenanthrenes did not affect the pI of α-tubulin neither in malignant nor in benign proliferating human cells (Figure 1B).

In view of the identified differences between human cancer and normal cells regarding NuMA and kinesins modification by the phenanthrenes, and in view of the major role of these proteins in mitosis [11, 16], we characterized the effects of the most potent phenanthrene PJ34 on mitosis of malignant versus benign cells.

Previous findings indicate the essential role of HSET/kifC1 in the spindle structure in human cancer cells [17–19, 21, 22]. HSET/kifC1 inhibition or silencing cause small aberrant spindles in human malignant cells [18, 19].

**Figure 2:**
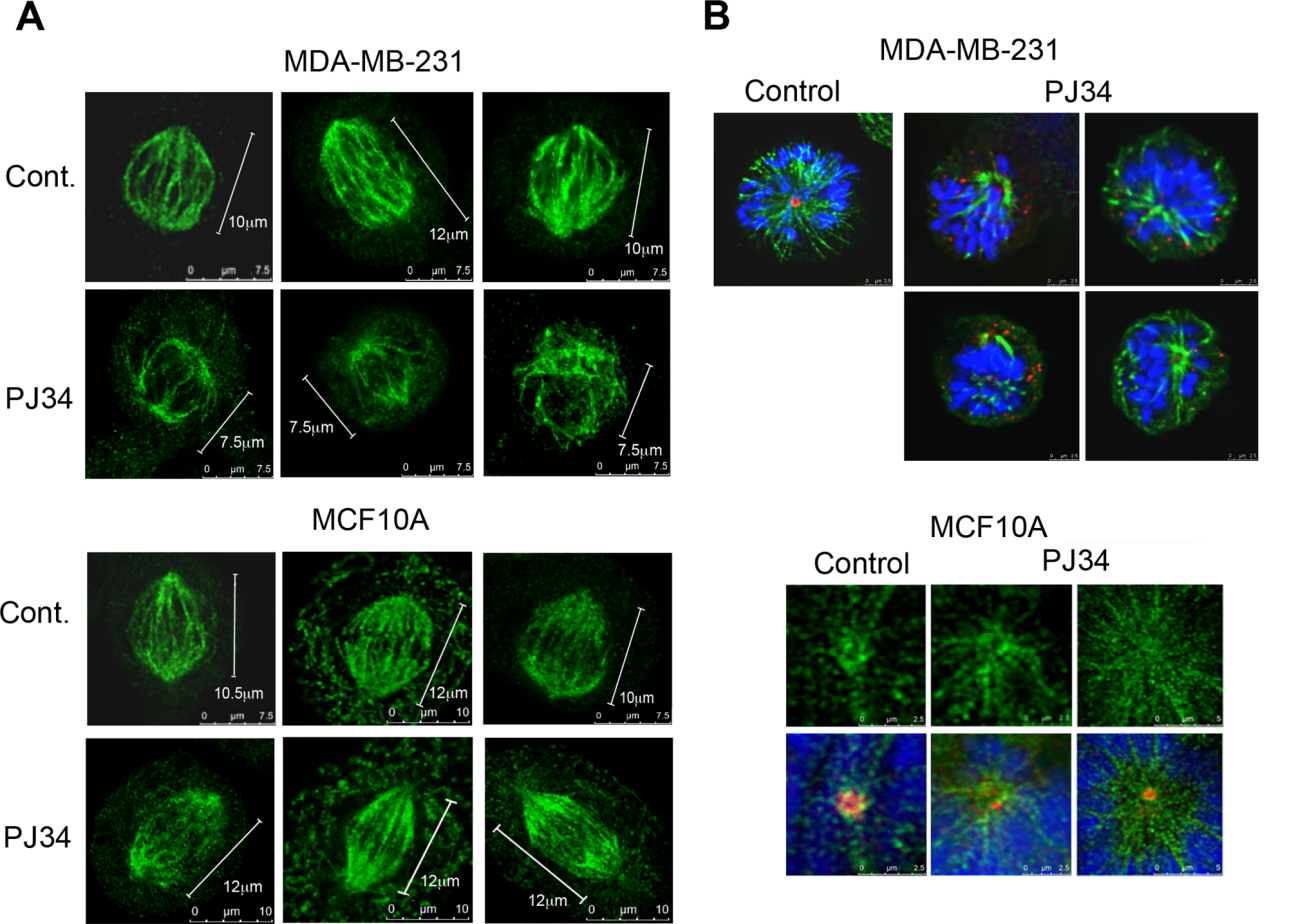
Aberrant spindles and impaired spindle poles in human cancer cells treated with the phenanthridine PJ34 (**A**) Small aberrant spindles were identified by confocal microscopy in randomly scanned human breast cancer MDA-MB-231 cells incubated with PJ34 (20 μM, 27 h). Spindles were immunolabeled for HSET/kifC1 (green) bound to microtubules. Similarly immunolabeled spindles are not impaired in PJ34 treated normal human breast epithelial cells MCF10A. Bars indicate the spindle length measured by the scale bar (*n* = 20; 3 different experiments), 95% of the spindles in MDA-MB-231 cells were shorter, 60 ± 5% in size. (**B**) Upper panel: Impaired spindle poles were identified in randomly scanned fixed MDA-MB-231 cells treated with PJ34 (20 μM, 27 h). Microtubules immunolabeled by α- tubulin (green), centrosomes immunolabeled by γ-tubulin (red) and chromosomes stained by DAPI (blue) are displayed. Lower panel: Spindle poles were not impaired in randomly scanned normal epithelial MCF10A cells treated with PJ34 (20 μM, 27 h). Microtubules identified by immunolabeled HSET14/kifC1 (green), centrosomes immunolabeled for γ-tubulin (red) and chromosomes stained by DAPI (blue) are displayed. The sampled spindles represent 95% of randomly scanned spindles (*n* = 20) of cancer MDA-MB-231 cells and all the randomly scanned spindles (*n* = 20) of normal epithelial MCF10A cells in 3 different experiments.

**Figure 3:**
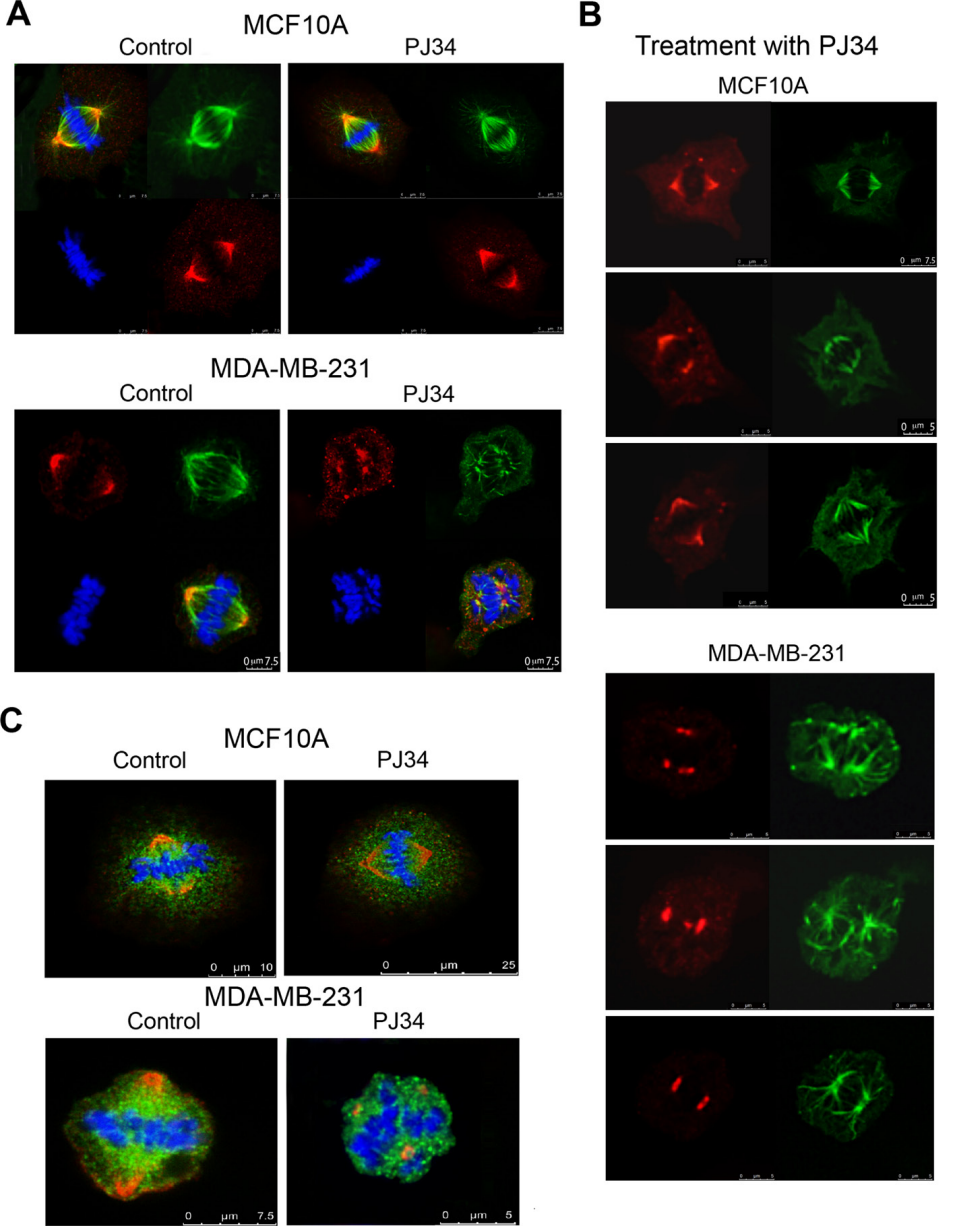
Impaired NuMA alignment in impaired spindles of human cancer cells treated with the phenanthridine PJ34 Scanned spindles in fixed human breast cancer MDA-MB-231 cells (upper frames in **A** and **B**) and normal breast epithelial MCF10A cells (lower frames in A and B), without or following treatment with PJ34 (20 μM, 27 h). Aberrant disorganized spindles, impaired NuMA alignment, and impaired chromosome alignments in the spindle mid-zone were observed only in cancer cells treated with PJ-34. Normal and malignant cells were immunolabeled for NuMA (red) and α-tubulin (green). Chromosomes are stained with DAPI (blue). (**C**) Scattered misaligned chromosomes and NuMA in disorganized spindles following PJ34 treatment (20 μM, 27 h) of MDA-MB-231 cancer cells. Proper alignment of chromosomes in the mid-zone of bifocal spindles and NuMA labeling in bifocal poles of PJ34 treated (20 μM, 27 h) MCF10A human epithelial cells. Both cell types were immunolabeled for NuMA (red) and kinesin HSET/kifC1 (green). Chromosomes are stained with DAPI (blue). Disorganized spindles and misaligned chromosomes were observed in 95% of spindles in PJ34 treated MDA-MB-231 cells (*n* = 20). Normal spindles, and chromosomes aligned in the spindle mid-zone were observed in all scanned PJ34 treated MCF10A cells (*n* = 20).

Similarly, smaller spindles were identified by confocal imaging in human malignant cells treated with PJ34 (20 μM, 27 h incubation), in comparison to the spindle size of untreated cells (60% ± 5% in size, in 95% of the 20 scanned spindles; Figure 2A). Immunolabeling of microtubules by α-tubulin or by HSET/kifC1 bound to the microtubules [17, 19–21] disclosed poorly constructed bundles and disorganized spindles in PJ34 treated cancer cells (90% of the 20 scanned spindles) (Figures 2 and 3). Treatment with PJ34 also disrupted the spindle poles (Figure 2B). Bi-focal spindles with clustered extra-centrosomes were not detected in the multi-centrosomal cells MDA-MB-231 [7, 8] (Figure 3A). PJ34 also altered NuMA localization, and the alignment of chromosomes in the spindle mid-zone (Figures 3 and Supplementary Figure 1). In PJ34 treated cancer cells chromosomes were scattered outside the spindle and close to the spindle poles (Figures 2B, 3A and 3C).

In contrast, PJ34 did not impair neither spindles and spindle poles, nor the bipolar localization of NuMA and the alignment of chromosomes in normal human epithelial cells MCF10A (*n* = 20; Figures 2 and 3).

Previous findings indicate a major contribution of both HSET/kifC1 and NuMA to the spindle poles structure [11, 16–18]. A major role of NuMA binding to microtubules, and its transfer along the microtubules to the spindle poles has been suggested to be crucial for the poles structure and stability [11–13, 29]. Furthermore, poles stability is required for proper alignment of chromosomes in the spindle mid-zone [29, 30]. In accordance, mutations in NuMA preventing its binding to microtubules, or a transient silencing of NuMA caused impaired spindle poles, un-segregated misaligned chromosomes and mitosis arrest [13, 30].

**Figure 4:**
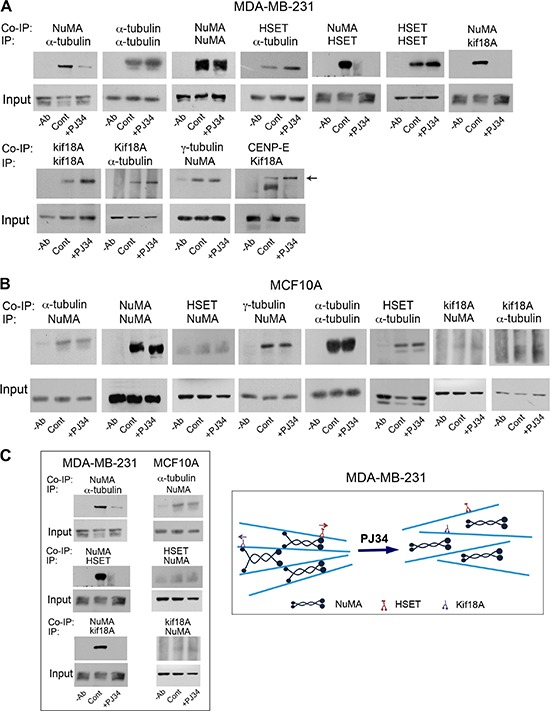
An impaired binding of NuMA to α-tubulin and to kinesins HSET/kifC1 and kif18A in human cancer cells treated with PJ34 NuMA was not co-immunoprecipitated with α-tubulin or kinesins HSET/kifC1 and kif18A in breast cancer cells MDA-MB-231 treated with PJ34 (20 μM, 27 h) (**A** and Supplementary Figure 2). No impaired binding of NuMA to α-tubulin and kinesins was detected in similarly treated human breast epithelial cells MCF10A (**B**). These results are schematically displayed in (**C**). Average 11.3 ± 1.1 times reduction in co-immunoprecipitated NuMA with α-tubulin was measured by scanning in PJ34 treated versus untreated MDA-MB-231 cancer cells. Average 1.2 ± 0.2 times reduction in the co-immunoprecipitated NuMA with α-tubulin was measured by scanning in PJ34 treated versus untreated normal epithelial cells MCF10A. Representative results of 4 different experiments are displayed.

In view of these results, a possible effect of the phenanthridine PJ34 on the binding of NuMA to kinesins and microtubules was examined in human cancer versus normal proliferating cells. Protein-to protein binding was tested by co-immunoprecipitation (Figure 4).

We found that the binding of NuMA to α-tubulin and to the kinesins HSET/kifC1 and kif18A was prevented in cancer cells treated with PJ34 (Figure 4A, 4C and Supplementary Figure 2). No similar effect on their binding was identified in normal epithelial cells (Figure 4B and 4C). In addition, PJ34 did not affect the binding of HSET/kifC1 and kif18A to α-tubulin in both cancer and normal cells (Figures 4A and B). This may reflect the binding of NuMA and kinesins to α-tubulin in the microtubules. PJ34 apparently prevents the binding of NuMA to microtubules and to kinesins HSET/kifC1 and kif18A, while HSET/kifC1 and kif18A binding to the microtubules is not affected. Changes in the kinesins movement along the microtubules were not excluded (Figure 4A). In addition, PJ34 did not interfere with kif18A binding to the motor protein CENP-E, a plus end-directed motor protein, functioning together with kif18A in kinetochore-microtubule capture during chromosome alignment in the spindle mid-zone [31] (Figure 4A).

**Figure 5:**
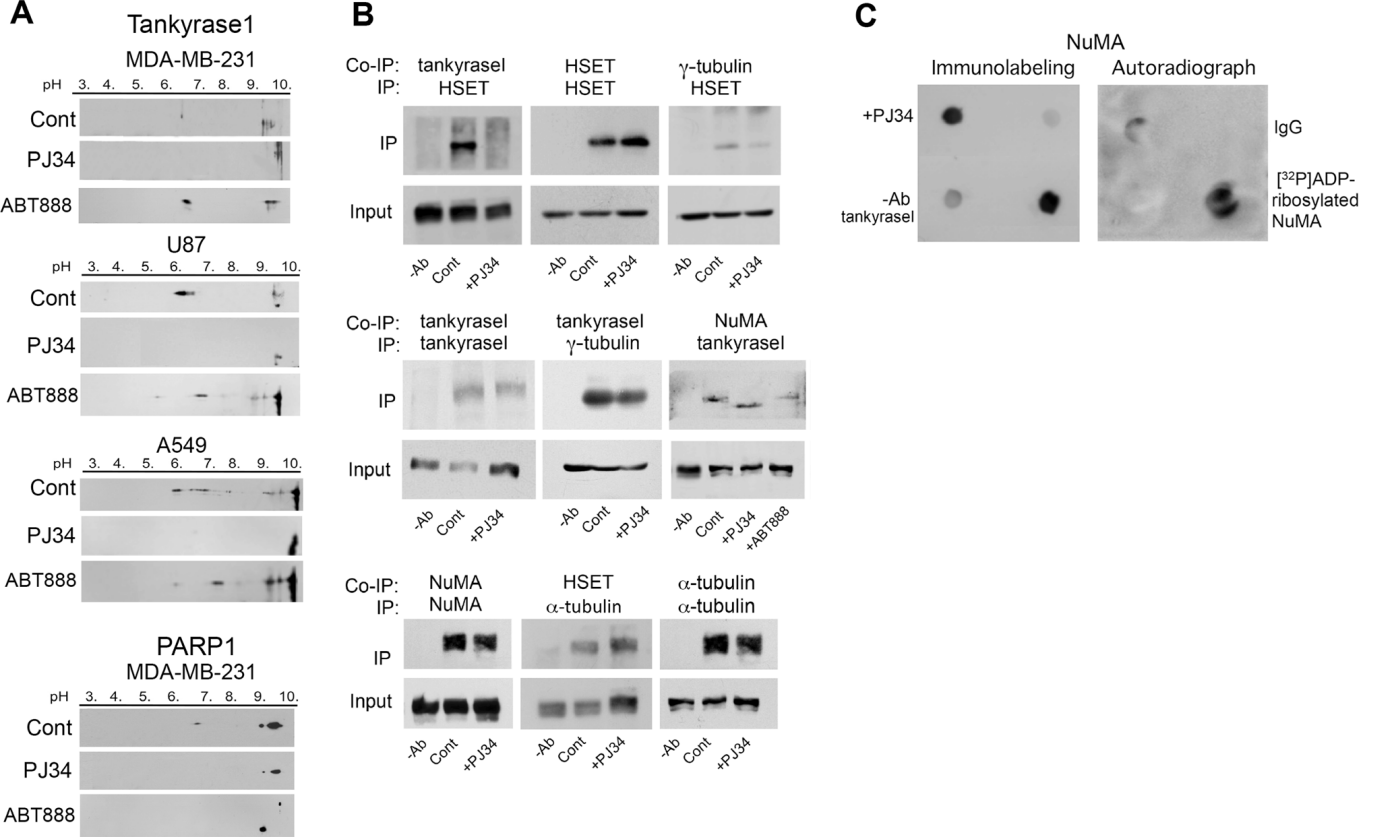
PJ34 inhibits NuMA and tankyrase1 polyADP-ribosylation in cancer cells (**A**) Inhibition of tankyrase1 modification measured by its shifted pI. Treatment with PJ34 (20 μM, 27 h) attenuated the shift in the isoelectric point of negatively charged tankyrase1 in lung cancer (A549), triple negative breast cancer (MDA-MB-231) and glioblastoma (U87) cancer cells. No similar shift of the isoelectric point of tankyrase1 was induced by the non-phenanthrene PARP1 inhibitor ABT888 (20 μM, 27 h). PolyADP-ribosylated PARP1 was similarly suppressed by both PARP1 inhibitors, PJ34 (20 μM) and ABT888 (20 μM). Representative results of 3 experiments in each cell type are displayed. (**B**) The binding of tankyrase1 to γ-tubulin or NuMA was measured by co-immunoprecipitation. Their binding in MDA-MB-231 cells was not affected by treatment with PJ34 (20 μM, 27 h). The binding of tankyrase1 to kinesin HSET/kifC1 was impaired. Representative results of 3 experiments are displayed. (**C**) [^32^P]polyADP-ribosylation of NuMA bound to tankyrase1 was inhibited after treatment of human cancer cells MDA-MB-231 with PJ34 (20 μM, 30 min). Binding of NuMA to tankyrase1 was measured by dot blot analysis (Methods). [^32^P]polyADP-ribosylated NuMA bound to tankyrase1 was trapped by tankyrase1 antibody on nitrocellulose membrane, auto-radiographed and immunolabeled. Results were repeated in 3 different experiments.

Since polyADP-ribosylation facilitates the binding of NuMA to proteins [14], prevention of NuMA binding to α-tubulin and kinesins in human cancer cells treated with PJ34 (Figures 4A), could be partially attributed to inhibition of tankyrase1 by the phenanthrenes PJ34, Phen and Tiq-A [14, 32, 33]. Their activity is not shared by non-phenanthrenes acting as potent PARP1 inhibitors [32, 33]. PJ34 (but not the non-phenanthrene potent PARP1 inhibitor ABT888) prevented the post-translational modification of tankyrase1 (apparently by polyADP-ribosylation, Figure 5A and 5C), without impairing the binding of tankyrase1 to its substrate NuMA (Figure 5B and 5C). NuMA co-immunoprecipitated with tankyrase1, either polyADP-ribosylated or not (Figure 5B and 5C). Also, the polyADP-ribosylation of NuMA was inhibited by PJ34 along with tankyrase1 inhibition. NuMA was not [^32^P]polyADP-ribosylated in cells treated with PJ34 (20 μM) (Figure 5C). No similar effect of PJ34 on NuMA binding to α-tubulin and kinesins due to tankyrase1 inhibition is expected in normal epithelial cells where tankyrase1 hardly exists [14, 15].

**Figure 6:**
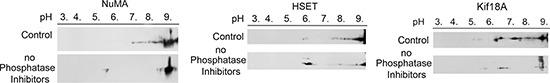
Phosphatase activity changes the isoelectric points of NuMA and kinesins HSET/kifC1 and kif18A in human breast cancer cells MDA-MB-231 In the absence of phosphatase inhibition enhancing kinase activity, some of the isoelectric points of NuMA and kinesins HSET/kifC1 and kif18A were shifted towards higher pH values (less negatively charged protein). Similar results were obtained in 3 different experiments.

However, NuMA modification by PJ34 is not attributed to its polyADP-ribosylation alone. In cancer cells, NuMA was similarly modified in the absence of phosphatase inhibitors (Figure 6), suggesting a possible role of kinase inhibition in the modification of NuMA by the phenanthrenes PJ34, Phen and TiqA (Figures 1E, 6). Also, phosphatase activity altered isoelectric points of kinesins HSET/kifC1 and kif18A similarly to their alteration by phenanthrenes (Figurers 1A, 1C and 6). Evidence indicating the control of their activity by phosphorylation [13, 16, 20, 22] support this notion. Although specific kinases inhibited by PJ34 were not identified, PJ34-induced inhibition of Pim1 could be one of the possible mechanisms implicated in NuMA modification by PJ34. The serine/threonine kinase Pim1 is highly expressed in cancer cells, it targets NuMA, and it is inhibited by PJ34 at the concentration range modifying NuMA in cancer cells [13, 34–36].

In cancer cells, tankyrase1, NuMA and HSET/kifC1 are localized in the spindle poles [15, 17–19], and tankyrase1 polyADP-ribosylation contributes to spindle poles stability [14, 15], possibly by inducing NuMA polyADP-ribosylation [14]. PJ34, inhibiting tankyrase1 polyADP-ribosylation ([32, 33]; Figure 5), interfered with the binding of tankyrase1 and NuMA to HSET/kifC1 in cancer cells (Figures 4A and 5B). On the other hand, tankyrase1, either polyADP-ribosylated or not, was bound to γ-tubulin, which is ubiquitous in the pericentriolar material [37, 38]. Thus, binding of tankyrase1 to centrosomes even outside the spindle poles is anticipated.

**Figure 7:**
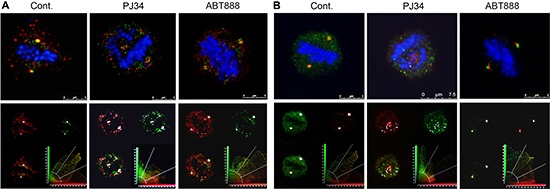
Co-localization of tankyrase1 with γ-tubulin and pericentrin in spindles of multi-centrosomal MDA-MB-231 cancer cells Bi-polar co-localization of tankyrase1 and γ-tubulin (**A**) or tankyrase1 and pericentrin (**B**) in spindles of untreated MDA-MB-231 cancer cells were identified by confocal microscopy scanning. Fixed cultured MDA-MB-231 cells were immunolabeled for tankyrase1 (green), γ-tubulin (red) and pericentrin (red). Chromosomes are stained by DAPI (blue). After incubation with PJ34 (20 μM, 27 h), the bipolar co-localization of tankyrase1 and γ-tubulin (A) or tankyrase1 and pericentrin (B) turned into multiple scattered foci of the co-localized proteins. Chromosomes were not aligned in the spindle mid-zone in all the PJ34 treated cells. Treatment with ABT888 (*n* = 20; 20 μM, 27 h) did not impair the bipolar co-localization of tankyrase1 and γ-tubulin (A), or tankyrase and pericentrin (B), nor impaired the chromosomes alignment in the spindle mid-zone in MDA-MB-231 cells. Co-localization of tankyrase1 and pericentrin, or tankyrase1 and γ-tubulin was detected in 95% of the randomly scanned spindles of both treated and untreated cells (*n* = 20).

Due to the tendency of un-polyADP-ribosylated tankyrase1 to form two-dimensional polymers that anchor proteins [15], and its tendency to bind γ-tubulin (Figure 5B), a possible anchorage of centrosomes to tankyrase polymers outside the spindle poles could promote extra-centrosomes de-clustering in multi-centrosomal cancer cells with disrupted spindle poles (Figure 2B). Thus, inhibition of tankyrase1 polyADP-ribosylation by PJ34, Phen or Tiq-A [32, 33] could both disrupt spindle poles (Figures 2B) and promote centrosomes de-clustering due to their binding to scattered tankyrase1 polymers ([15], Figures 5 and 7).

Bi-focal convergence points of tankyrase1 co-localized with γ-tubulin and pericentrin were identified by confocal scanning microscopy in the spindle poles of multi-centrosomal human cancer cells (Figure 7A and 7B). However, this bi-focal co-localization of tankyrase1 with both γ-tubulin and pericentrin was prevented by treatment with PJ34. Instead, tankyrase1 co-localized with both γ-tubulin and pericentrin in scattered clusters in 95% of the PJ34 treated cells (Figure 7A and 7B). Notably, the potent PARP1 inhibitor ABT888, which does not inhibit tankyrase1 ( [32]; Figure 5A), and did not affect the pI of NuMA in cancer cells (Figure 1E) - did not interfere with the bi-focal co-localization of tankyrase1 with γ-tubulin and pericentrin in the spindle poles (Figure 7A and 7B). These results are in accordance with previously reported no-interference of ABT888 with extra-centrosomes bi-focal clustering in multi-centrosomal cancer cells [7, 8], and they support a PARP1-independent activity of the phenanthrenes in extra-centrosomes de-clustering (Figures 1, 5A).

Stable spindle poles are required for normal alignment of chromosomes in the spindle mid-zone [29, 30]. A normal transfer of NuMA along the spindle microtubules towards the spindle poles is required for their construction and stability (11–14). Thus, an impaired binding of NuMA to α-tubulin and kinesins HSET/kifC1 and kif18A in cancer cells treated with PJ34 (Figure 4A and 4C) could impair the formation of stable spindle poles [11, 17, 21], and the alignment of chromosomes in the spindle mid-zone [29, 30]. This was assessed by confocal imaging of PJ34 treated cancer cells (Figures 2, 3, 7). Chromosomes alignment in the mid-zone was impaired in abnormal spindles with disorganized spindle poles in cancer cells treated with PJ34 [18, 21, 29, 30]. Misaligned chromosomes cause mitotic cell-death irrespectively of extra-centrosomes de-clustering [6, 18, 41]. So, mitotic cell death might not be the consequence of un-clustered extra-centrosomes [7, 8]. Un-clustered centrosomes could be the consequence of disorganized spindle poles in multi-centrosomal cancer cells treated with PJ34 (Figures 2B, 3, 7).

Notably, the impaired binding of modified NuMA to microtubules and to modified kinesins HSET/kifC1 and kif18A in human cancer cells, correlated with the exclusive destruction of spindles and spindle poles in these cells. PJ34 did not interfere with the modifications of NuMA and kinesins in normal proliferating cells, nor impaired their spindles (Figures 1, 2, 3, 4).

Thus, phenanthrenes inhibiting polyADP-ribosylation and phosphorylation of NuMA and kinesins HSET/kifC1 and kif18A in human cancer cells (Figures 1, 4, 5, 6) may exclusively interfere with their activity in spindle construction (Figures 2, 3 and 7), and cause mitotic arrest and cell death [6–8, 39, 40]. A possible implication of such exclusive spindle destruction during mitosis in human cancer cells on tumor growth arrest was examined in human malignant tumors treated with PJ34.

**Figure 8:**
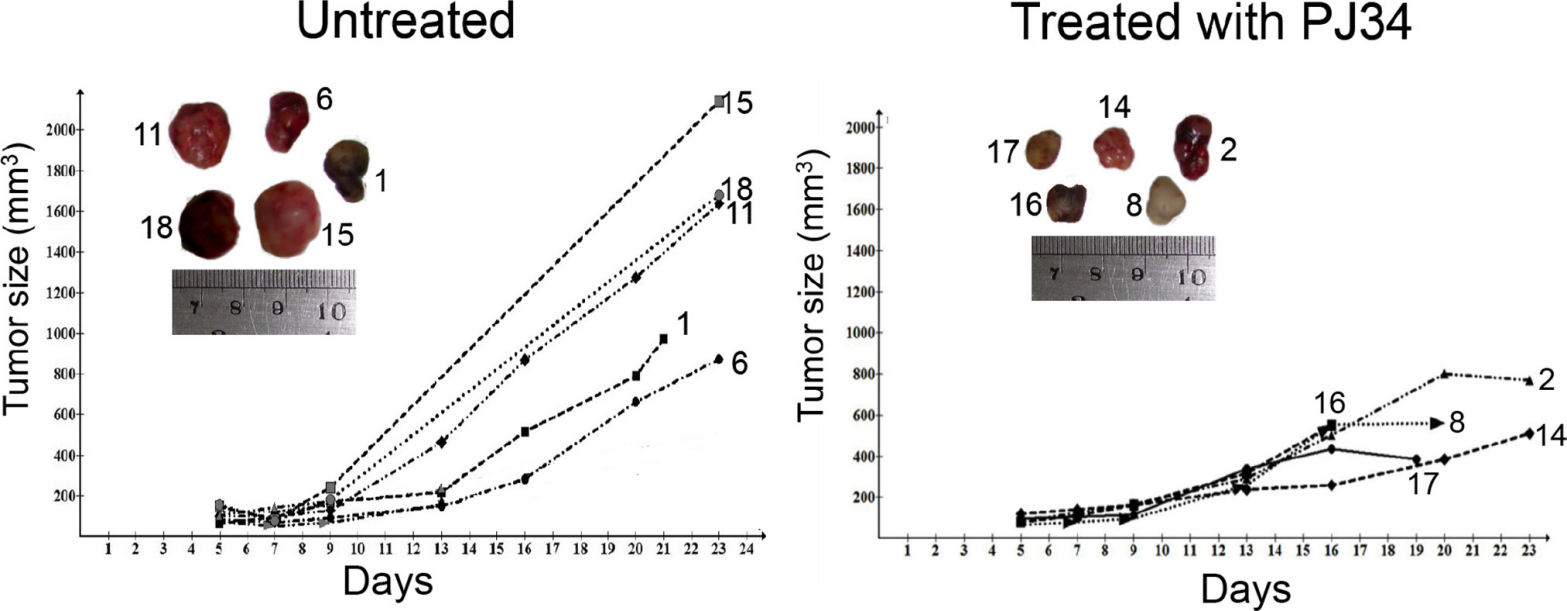
Human tumors growth arrested in nude athymic mice treated with PJ34 The growth of human triple negative breast MDA-MB-231 xenotransplants was significantly arrested in the indicated nude athymic mice injected i.p. (intraperitoneal) with PJ34 (60 mg/kg, daily for 14 days). The significance of the change in tumor growth rate was calculated by using one-tailed *t-test* for estimating the statistical significance of the difference between the group means, without assuming equal variance. The result was statistically significant: *p* = 0.036 (*t* = 2.14, df = 6.33). The daily measured average tumor size in each group is presented in Supplementary Figure 3.

We measured the effect of PJ34 on the growth of rapidly developing tumors of human triple negative breast cancer MDA-MB-231 xenotransplants in athymic nude mice (Figure 8). In these experiments, mice were injected sub-cutaneously with 5 × 10^6^ MDA-MB-231cells/animal. Localized tumors (approximately 0.1 cm^3^) were developed within 4–5 days. These tumors rapidly grew during the next 18 days in the group of un-treated mice (*n* = 5). In contrast, tumor growth was arrested in the group of mice (*n* = 5) treated with PJ34 (60 mg/kg, injected intra-peritoneal, daily for 14 days) (Figure 8, Supplementary Figure 3). These results suggest a possible efficacy of PJ34 treatment in cancer therapy by activation of a fast cell-death mechanism during mitosis in cancer cells, without affecting normal cells.

## MATERIALS AND METHODS

Cell cultures Human triple-negative breast cancer cell line MDA-MB-231 and cell-lines of glioblastoma U87, non-small lung cancer A549 and pancreas cancer PANC1, as well as human epithelial cells MCF-10A, were supplied by ATCC Co. (American Type Culture Collection, P.O. Box 1549, Manassas, VA 20108, USA. In Israel, Almog Diagnostic & Medical equipment Ltd). Human cancer cells were cultured in 6-wells multi-dish plates (Nunc Denmark). The human malignant cells were maintained in a medium containing DMEM (cat # 01-055-1A) 10% fetal bovine serum (FBS) (cat # 04-121-1A), 1% L-Glutamine (cat # 03-020-1B), and 1% Pen-Strep Ampho (cat # 03-033-1B), purchased from biological industries, Israel. Human epithelial cells MCF10A were cultured in DMEM/F12 (Gibco, Cat No 31330) with FBS (Gibco) containing: 5%, EGF (100 μg/ml, Cytolab) 0.02%, Hydrocortisone (50 μM, Sigma) 1%, Insulin (10 mg/ml, Sigma) 0.1%, Pen/Strep (biological industries) 1%.

Antibodies included antibodies directed against HSET/kifC1 (polyclonal rabbit Cat 12313, Cell Signaling technology, or monoclonal mouse (IgG2a) Cat sc-100947), kif18A (polyclonal Rabbit (cat 19245-1-AP, ProteinTech), NuMA (Rabbit polyclonal Cat # A301-509A Bethyl Lab), γ-tubulin (Sigma,T5192), α-tubulin (Sigma, T9026 and Santa Cruz Biotech., sc-5286 lgG2b) and pericentrin (Abcam ab4448). Antibodies directed against tankyrase1 (Rα Tank1 762) were donated by Prof Susan Smith, NYU [14].

Treatments of cultured cells. We examined the effect of phenanthrenes acting also as PARP inhibitors. These included PJ34, (N-(6-Oxo-5,6-di hydrophenanthridin-2-yl)N,N-dimethyl-acetamide), Tiq-A, (4H-Thieno[2,3-c] isoquinolin -5-one) (Sigma Israel) and Phen, (6(5H)-phenanthridinone). PJ34 and Phen were purchased from Alexis Biochemicals, (Alexis Corporation, Lausen, Switzerland). The non-phenanthrene ABT888 (2-[R-2methylpyrrolidin-2-yl]-1H-benzimidazole-4-carboxamide, was purchased from Abbott Labs, Illinois, U.S.A

Protein extraction for two-dimensional electrophoresis TNE buffer was used for cells lysis, it contains: 150 mM NaCl, 1 mM EDTA pH8.0, 20 mM Tris-HCl pH8.0, 1% NP-40, phosphatase inhibitors (2 mM sodium orthopvanadate and 25 mM β-glycerolphosphate) [42] and protease inhibitors cocktail (Sigma) 2%. Cells were homogenized in the TNE buffer (about 1ml of buffer to 170 mg of wet weight of cell) and incubated for 1 h on ice. Suspensions were pelleted at 8000 g for 15 min at 4^°^C. Proteins in the supernatants were precipitated with methanol (added 9 volums to the sups, 30 min, RT), and dissolved in 9.5M Urea and 2% CHAPS.

Two-dimensional (2-D) gel electrophoresis was used to identify covalent post translational modifications of proteins that cause changes in their isoelectric point (pI). In the first dimension, samples (350 μg protein per 100 μl sample) were mounted on Immobiline DryStrip kit (Amersham Biosciences, Uppsala, Sweden) [26], and separated by pH gradient. In the second dimension the mounted proteins are separated by size in polyacrylamide slab gels. Shifted pI of a protein towards lower pH indicates its enhanced negative charge (e.g., by phosphorylation or polyADP-ribosylation). This method has been used before to identify activated G-proteins and polyADP-ribosylated PARP1 [27, 28].

Co-immunoprecipitation was used to identify binding between proteins in whole cell protein extracts. We followed previously reported methods [14]. Cells were dissolved in TNE buffer (1 h on ice). Suspensions were centrifuged (8000 g for 15 min at 4^°^C). Proteins in the supernatants were used for immunoprecipitation [14]. A specific antibody bound to protein A conjugated to agarose beads was used to immunoprecipitate the target protein. The beads with the bound proteins were discarded after washing in TNE buffer and boiled (1 min) in twice concentrated Laemmli sample buffer. The bound proteins were separated on SDS-PAGE and immunolabeled.

Immunocytochemistry and confocal microscope imaging. Fluorescent Immuno-labeled proteins were scanned by confocal microscopy in cells fixed after treatments, as described before [8]. Spindle filaments were labeled with antibodies directed against α-tubulin in the microtubules (monoclonal T9026; Sigma), or by antibodies directed against HSET/kifC1 attached to the filaments. Centrosomes were labeled with antibodies directed against γ-tubulin or pericentrin.

Dot Blots. This method was described before [43]. We used this method to identify [^32^P]polyADP-ribosylated NuMA in lyzed MDA-MB-231 cells, without or after treatment with PJ34 ([^32^P]NAD (800 Ci/mmol; 1 μCi/sample; PerkinElmer, Hod Hasharon, Israel)

[^32^P]PolyADP-ribosylated NuMA bound to antibody directed against tankyrase1 was anchored to nitrocellulose membranes. The blots were blocked in ‘binding buffer’ (50 mM Tris-HCl pH7.5, 120 mM NaCl, 0.1% NP40, 0.5 mM PMSF, 20 mg/ml BSA) for 30 min, RT. After ‘blocking’ blots were incubated for 3 h at RT in ‘binding buffer’ containing the [^32^P]polyADP-ribosylated proteins and antibody directed against tankyrase1 (Rα Tank1 762), and washed with TBS-Tween-20 0.1%. Anchored [^32^P]PolyADP-ribosylated NuMA was detected by autoradiography and immunolabeling of the nitrocellulose membranes.

*In*-*vivo* experiments: MDA-MB-231 cells were injected subcutaneously in Hanks’ balanced solution (HBSS) at concentration 5 × 10^6^ cells/animal (50 × 10^6^ cells/ml; 97% viability tested by trypan blue staining) in ratio 2:1 mix with Matrigel™/PBS mix (1:1 ratio). PJ34 (60 mg/kg) was injected intra-peritoneal, daily for 14 days after tumors growth (0.1 mm^3^). Tumor growth was measured during the treatment with PJ34. The experiments were performed by Harlan (Envigo, Jerusalem). All experimental protocols were approved by the Animal Care and Use Committees of the Ministry of Health, Israel.

## SUPPLEMENTARY MATERIALS FIGURES AND TABLES


