# Correction: Exclusive destruction of mitotic spindles in human cancer cells

**DOI:** 10.18632/oncotarget.27499

**Published:** 2020-04-07

**Authors:** Leonid Visochek, Asher Castiel, Leonid Mittelman, Michael Elkin, Dikla Atias, Talia Golan, Shai Izraeli, Tamar Peretz, Malka Cohen-Armon

**Affiliations:** ^1^The Neufeld Cardiac Research Institute, Department of Physiology and Pharmacology, Sackler School of Medicine, Tel-Aviv University, Tel-Aviv 69978, Israel; ^2^Cancer Research Center, Sheba Medical Center, Ramat Gan 53621, Israel; ^3^The Imaging Unit, Sackler School of Medicine, Tel-Aviv University, Tel-Aviv 69978, Israel; ^4^Sharett Oncology Institute, Hadassah Medical Center, Ein-Kerem, Jerusalem 91120, Israel; ^5^The Department of Human Molecular Genetics and Biochemistry, Sackler School of Medicine, Tel-Aviv University, Tel-Aviv 69978, Israel; ^6^Sagol School of Neuroscience, Tel-Aviv University, Tel-Aviv 69978, Israel

**This article has been corrected:** There was an error during the assembly of the bottom panel of Figure 5B. Instead of displaying IP of α-tubulin by α-tubulin and NuMA IP by NuMA, NuMA IP by NuMA was displayed twice. The correct Figure 5B is shown below. There are no changes in the figure legend. The authors declare that this correction does not change the results and conclusions of the paper.

Original article: Oncotarget. 2017; 8:20813–20824. 20813-20824. https://doi.org/10.18632/oncotarget.15343

**Figure 5:**
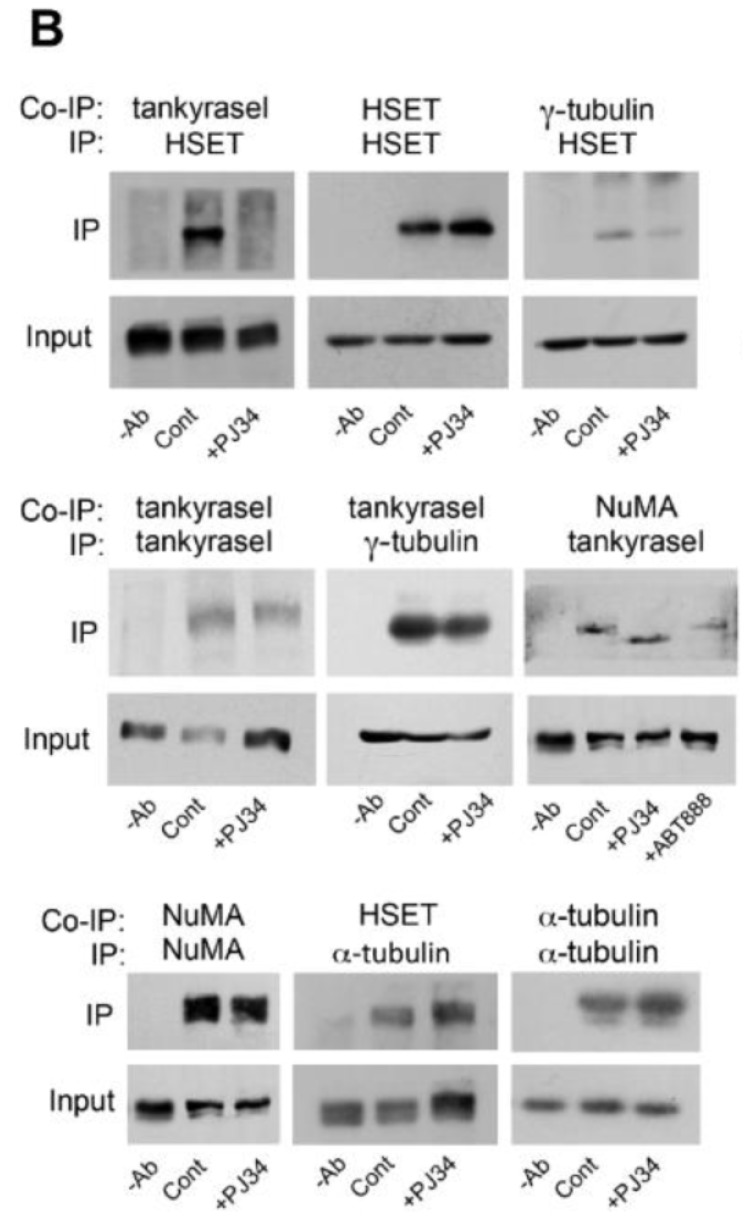
PJ34 inhibits NuMA and tankyrase1 polyADP-ribosylation in cancer cells. (**B**) The binding of tankyrase1 to γ-tubulin or NuMA was measured by co-immunoprecipitation. Their binding in MDA-MB-231 cells was not affected by treatment with PJ34 (20 μM, 27 h). The binding of tankyrase1 to kinesin HSET/kifC1 was impaired. Representative results of 3 experiments are displayed.

