# Supplementary material for: Exclusive destruction of mitotic spindles in human cancer cells
Source: Oncotarget. 2017 Feb 15;8(13):20813–24. doi: 10.18632/oncotarget.15343 (PMC5400547; doi:10.18632/oncotarget.15343)
Supplement: Supplementary file 1 [file oncotarget-08-20813-s001.pdf]

Exclusive destruction of mitotic spindles in human cancer cells

Supplementary Materials

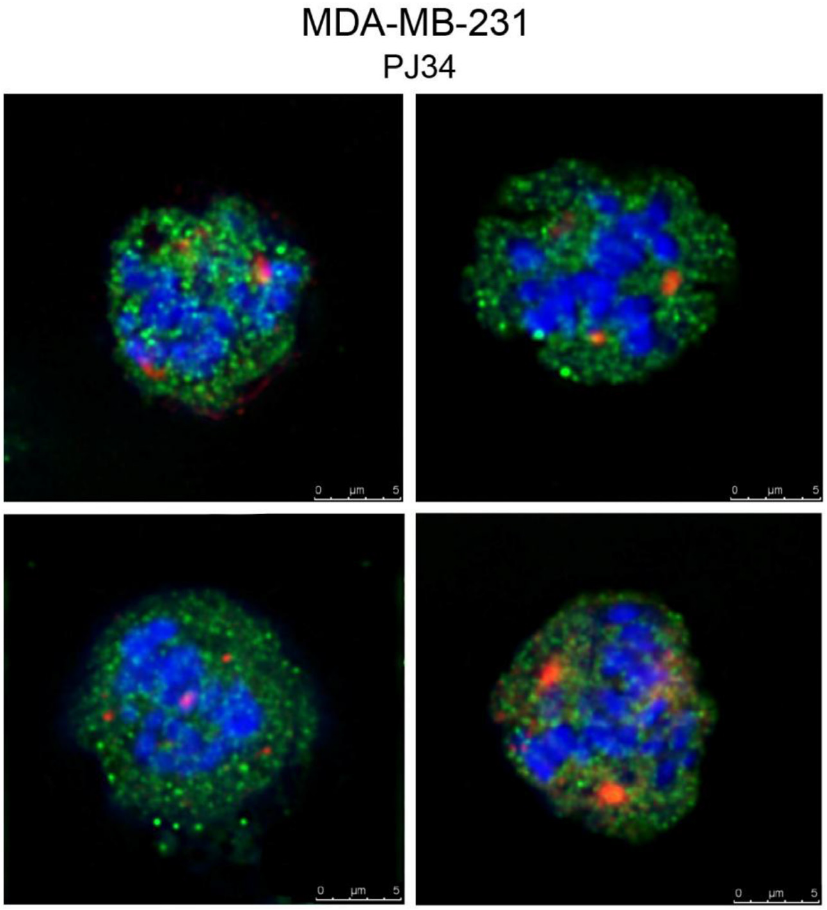

Supplementary Figure 1: (Supplementary to Figure 3C) Disorganized spindles with scattered misaligned chromosomes and multiple foci of NuMA in MDA-MB-231 cancer cells treated with PJ34 (20 µM, 27 h). NuMA (red), kinesin HSET/kifC1 (green), Chromosomes stained with DAPI (blue).

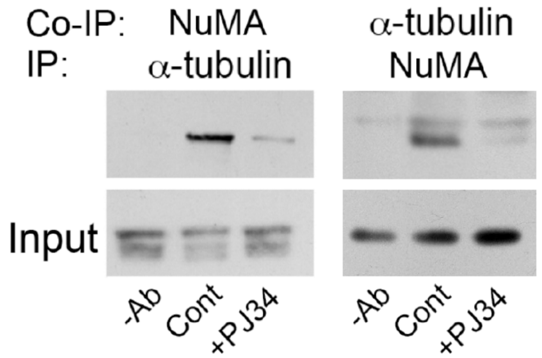

Supplementary Figure 2: (Supplementary to Figure 4A) Co-immunoprecipitation of NuMA by α-tubulin and of α-tubulin by NuMA prevented in MDA-MB-231 cells treated with PJ34 (20 µM, 27 h).

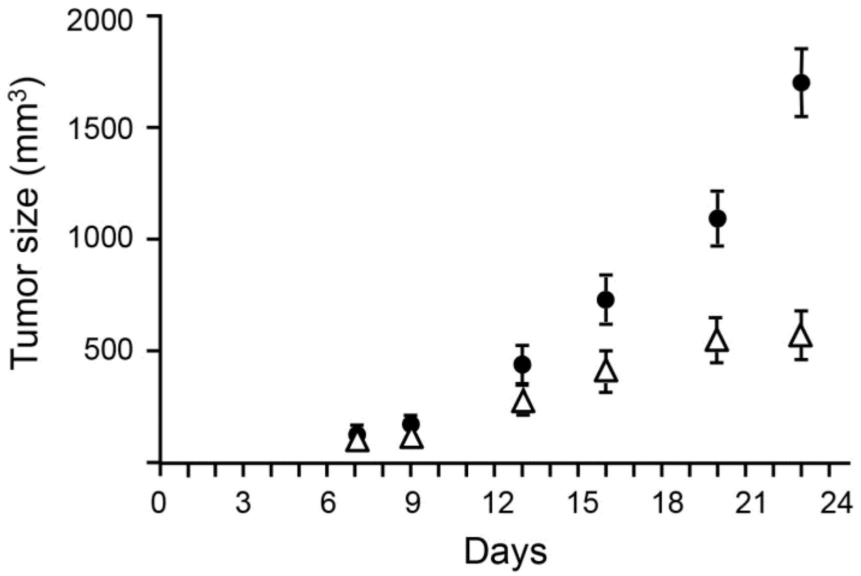

Untreated		Treated	
Tumor size	± Δ	Tumor size	± Δ
1782	232.8	639	92.3
1090	137.7	594	107.4
714	125	460	49
386	71.6	287	16.2
154	22.2	140	11.
130	11.	110	9.4

Supplementary Figure 3: (Supplementary to Figure 8) Average tumor size developed in athymic mice injected subcutaneously with human triple-negative breast cancer cells MDA-MB-231 (5 × 10^6 cells/animal, 97% viability tested by trypan blue staining, were injected in ratio 2:1 mix with Matrigel™/PBS mix (1:1 ratio)), without or during treatment with PJ34 (60 mg/kg, intra-peritoneal injection, daily for 14 days).
